# Breast cancer awareness among women in Eastern China: a cross-sectional study

**DOI:** 10.1186/1471-2458-14-1004

**Published:** 2014-09-26

**Authors:** Li-Yuan Liu, Fei Wang, Li-Xiang Yu, Zhong-Bing Ma, Qiang Zhang, De-Zong Gao, Yu-Yang Li, Liang Li, Zhong-Tang Zhao, Zhi-Gang Yu

**Affiliations:** Epidemiology Institute, School of Public Health, Shandong University, Jinan, Shandong China; Department of Breast Surgery, The Second Hospital of Shandong University, Jinan, 250033 Shandong China

**Keywords:** Breast cancer, Knowledge, Chinese women, Cancer awareness

## Abstract

**Background:**

High breast cancer mortality has been attributed to lack of public awareness, which leads to late diagnoses. As little is known about the level of knowledge and awareness of breast cancer in China, this study was designed to explore it among women in Eastern China.

**Methods:**

We conducted a cross-sectional survey covering 122,058 females around Shandong, Hebei, Jiangsu and Tianjin, in Eastern China, using in-person interviews based on a self-designed structured questionnaire. Student’s *t*-test, Pearson’s *χ*^2^ test, reliability analysis, exploratory factor analysis, univariate and multivariate logistic regression analyses were performed in the statistical analysis.

**Results:**

The results showed poor awareness of breast cancer among women aged 25–70 years in Eastern China. Only 18.6% of women were highly aware in the study, whereas 81.4% were poorly aware. Among all participants, family history of breast cancer was the best accepted risk factor for breast cancer (awareness rate 31.5%), followed by menarche at age before 12 (11.2%), no parity or late childbirth (13.9%), menopause at a late age (13.7%), high-fat diets (19.1%), long time drinking (19.5%) and long-term use of estrogen drugs (20.7%). Multivariate logistic regression analysis (*α* = 0.05) identified nine variables that predicted awareness of breast cancer: age (OR = 0.975, 95% CI: 0.960–0.990), location (OR = 1.675, 95% CI: 1.602–1.752), occupation (OR = 4.774, 95% CI: 4.316–5.281), family history of breast cancer (OR = 1.234, 95% CI: 1.073–1.420), household annual income (OR = 0.418, 95% CI: 0.400–0.436), behavioral prevention score (OR = 4.137, 95% CI: 3.991–4.290), no smoking (OR = 2.113, 95% CI: 1.488–2.999), no drinking (OR = 1.427, 95% CI: 1.018–2.000), overall life satisfaction (OR = 0.707, 95% CI: 0.683–0.731).

**Conclusions:**

Our study indicates insufficient awareness of breast cancer among women in Eastern China, and an urgent need for health education programs on this subject.

## Background

Breast cancer is the leading cause of cancer death in women worldwide by far. About 1.67 million new cases were diagnosed, and 522,000 deaths from breast cancer occurred in 2012 [1]. Because of increased life expectancy and urbanization, the incidence of breast cancer has been rising steadily in both developed and developing countries. According to the American Cancer Statistics Report, an estimated 232,340 new cases of invasive breast cancer were diagnosed in the US during 2013, accounting for 29% of new cancer cases among women [2]. In China, the crude incidence was estimated to be 42.55 per 100,000 women [3]. Based on National Central Cancer Registry 2009, breast cancer is the most frequently diagnosed cancer among Chinese women, irrespective of age group or ethnicity, accounting for 16.18% of all cancer types [3].

Early diagnosis of breast cancer has been clearly shown to reduce mortality and improve survival [4–6]. Combined results from randomized screening trials suggest that mammography reduces the risk of dying from breast cancer by 15–20%, and studies of modern mammography screening programs in Europe found that the risk of breast cancer death was reduced by more than one-third [7–11]. According to the *Breast Cancer Facts & Figures 2013–2014* report, 5-year relative survival decreases with more advanced stage at diagnosis, at 99% for localized disease, 84% for regional disease, and 24% for distant-stage disease [12]. Larger tumor size at diagnosis is also associated with decreased 5-year relative survival, with 95% for tumors ≤ 2.0 cm, 83% for tumors 2.1–5.0 cm, and 65% for tumors >5.0 cm among women with regional disease [5]. Breast cancer screening is an effective way to detect early-stage breast cancer [13, 14]. However, China has no such nationwide screening program for breast cancer at present. Barriers to implementation of a population-based mammography screening program include insufficiently convincing cost-effectiveness data; the large, widely dispersed population; insufficient mammography equipment; and inadequate insurance coverage for such a program [15]. Lucky to see National Health and Family Planning Commission of China began to carry out the relevant screening program since 2010. For example, “Two-cancer”(cervical cancer and breast cancer) screening in rural women in China [16].

Studies from developed countries showed that attitude and orientation of healthcare providers are important determinants of use of breast-screening programs [17, 18]. Promotion of public health measures also requires that both health-care workers and general public have appropriate knowledge, attitude and beliefs concerning the behavior being promoted [5]. Thus, increasing comprehensive knowledge and awareness of breast cancer could facilitate breast self-examination (BSE) and mammography screening. However, poor awareness and knowledge about breast cancer symptoms and screening methods has been previously reported by several different studies [14, 19, 20].

To plan critically needed breast cancer awareness and education programs that optimally address Chinese women, healthcare professionals and planners must know their current level of understanding. Therefore, we performed this cross-sectional survey in Eastern China to explore public awareness of breast cancer-related symptoms and risk factors.

## Methods

### Study subjects

All participants were selected from an earlier epidemiological survey, funded by the Ministry of Health of the People’s Republic of China, of 122,058 women aged 25–70 years, from Shandong, Hebei, Jiangsu and Tianjin. The cross-sectional study used multi-stage stratified and cluster sampling methods. The target population covered women aged 25–70 years of the Han ethnic group, who had ≥ 2 cumulative years of local residence and ≥ 6 months of local residence at the time of survey. Long-term migrant workers, who had left their hometowns for work, were excluded from this study. Provinces in Eastern China where the Han ethnic group mainly resides, including Shandong, Jiangsu, Hebei and Tianjin, were selected for the survey. We then randomly selected counties or regions from each province, and randomly selected villages or communities from the selected counties or regions. Women who met the study requirements were then selected for the survey.

### Study design

This study was a cross-sectional epidemiological survey that included face-to-face interviews and breast examinations. Data were collected through in-person interviews based on a self-designed structured questionnaire. The questionnaire covered six areas gathered from participant interviews: (1) demographic characteristics such as age, marital status, education, occupation, etc.; (2) female physiological and reproductive factors: menstrual cycle history, childbearing history, etc.; (3) medical and family history, including breast-related diseases and family history of breast cancer; (4) dietary habits: frequency of the intake of various types of food; (5) lifestyle habits including smoking, alcohol intake, and psychological conditions (the items under psychological status were summed to calculate the overall life satisfaction scores); (6) knowledge of risk factors for breast cancer, early signs and symptoms of disease (cumulative scores of these relevant items were counted as the related knowledge score and behavioral prevention score). Except for the basic demographic information, all questions had multiple-choice responses (e.g., yes/no or 1/2/3/4), and the answer choices were categorized and quantified when possible. For all variables covered by the questionnaire, the answers were defined by strict criteria.

### Implementation

This study was approved by the ethics committees of the Second Hospital of Shandong University and those at the collaborating institutions in each region and informed consent was obtained from all the study subjects. However, although all the official seals and signatures of committee members are available, this study has no ethics reference number. As a cross-sectional study, all interviews were completed over a 2-month period, July 15–Sept 15, 2008. Surveyors and physicians were recruited in each survey region before implementing this study. To ensure objectivity and authenticity, all participating investigators, and the physicians who perform physical examinations received strict and standardized professional training before beginning the project. A total of 77 physicians and 248 surveyors participated in the on-site survey process.

### Scoring scheme

Knowledge of breast cancer was assessed by requesting the respondents to answer 15 items included in the questionnaire on symptoms and risk factors of breast cancer (Table 1). Thereafter, each correct response (“yes”) was scored one (1) point and each wrong response (“no” or “do not know”) was scored zero (0). Total scores thus ranged from 0 to 15. Finally, the total score was transformed into a centesimal system. Respondents with scores 0–60 were considered to have poor knowledge in this study, whereas those with 61–100 points were considered to have good knowledge. Behavioral prevention was cumulative scored by five items including participation in breast cancer screening, BSE, and clinical breast examination (CBE), for which subjects received 1 point each if they took part, for total scores of 0–5. The overall life satisfaction score was cumulatively based on 12 items for which high scores indicated low life satisfaction, and low scores indicated high life satisfaction.

**Table 1:** **Summary of responses by participants to breast cancer awareness questionnaire (** ***N*** **= 122,058)**

***Questions***	***N***	%
**Do you know breast cancer**		
Yes	98362	80.6
No	23696	19.4
**Do you think screening is helpful for early detection of breast cancer**		
Yes	86405	70.8
No	35653	29.2
**Do you think the early detection of breast cancer can improve survival**		
Yes	86165	70.6
No	35893	29.4
**Knowledge about breast symptoms**		
**Local discomfort in breast**		
Yes	34922	28.6
No	22939	18.8
Don’t know	63965	52.4
**Lump in breast**		
Yes	49812	40.8
No	13601	11.1
Don’t know	58407	47.9
**Axillary nodes**		
Yes	33658	27.6
No	12863	10.5
Don’t know	75266	61.7
**Nipple retraction**		
Yes	25954	21.3
No	15452	12.7
Don’t know	80394	65.9
**Nipple discharge liquid**		
Yes	28084	23
No	13378	11
Don’t know	80330	65.8
**Related factors of breast cancer**		
**Menarche at age before 12**		
Yes	13727	11.2
No	18247	14.9
Don’t know	89824	73.6
**No parity or late childbirth**		
Yes	16932	13.9
No	16209	13.3
Don’t know	88652	72.6
**Menopause at a late age**		
Yes	16758	13.7
No	15644	12.8
Don’t know	89373	73.2
**Long time drinking**		
Yes	23793	19.5
No	12827	10.5
Don’t know	85160	69.7
**High-fat diets**		
Yes	23347	19.1
No	12628	10.3
Don’t know	85819	70.3
**Long-term use of estrogen drugs**		
Yes	25231	20.7
No	9885	8.1
Don’t know	86663	71
**Family history of breast cancer**		
Yes	38479	31.5
No	10591	8.7
Don’t know	72704	59.5
**Awareness of breast cancer**		
Highly aware	22740	18.6
Poorly aware	99318	81.4

### Quality control

All interviews in this survey were completed within 2 months. All survey subjects were selected strictly according to the random sampling method. During the survey, a supervisor in each region oversaw the surveyors and verified their work; and 10% of the questionnaires completed each day were randomly inspected for completeness, accuracy and standardization. After the quality assessment was completed, all identified errors and missed survey items were promptly corrected. If some information was missed in the questionnaire, the interviewer would collect the missing information from the original survey site the next day. Finally, upon survey completion, samples were re-screened for each survey site; results showed 92.19% consistency. Standardized training was provided to the data entry personnel; double data entry was also performed.

### Statistical analysis

The database was established using the software EpiData3.1. Statistical methods, including Student’s *t*-test, Pearson’s *χ*^2^ test, reliability analyses, exploratory factor analysis, and univariate and multivariate logistic regression analyses, were used to identify factors related to knowledge of breast cancer. For all outcomes, demographic variables for which *P* <0.05 in univariate analyses were entered into the multivariate model, and retained in the final model if *P* < 0.05. Odds ratios (OR) with 95% confidence intervals were also calculated. All data analyses were performed using SPSS13.0.

## Results

This epidemiological survey initially selected 147,538 women, of whom 124,758 were reached to complete the survey, giving a loss rate of 15.44%. Of the 124,758 women surveyed, 1037 subjects were younger than 25 years, and 1629 subjects were older than 70 years. A further 34 were excluded owing to unavailability of important information. Thus, 122,058 women were included in our final analysis.

Table 2 shows their demographic characteristics. Their mean age was 44.2 ± 11.6 years; 88,362 (72.39%) were from rural areas and 33,696 (27.61%) from urban areas; 67,827 (55.6%) were peasants, 21,311 (17.5%) workers, 2376 (1.9%) medical staff, and 30,544 (25.0%) had other occupations (e.g., teacher, driver, civil servant, individual business, housewife, etc.); 117,142 (96.0%) were married, 1464 (1.2%) single and 3452 (2.8%) widowed or divorced; 39.9% finished primary school or had less education, 34.8% finished middle school, 15.7% finished high school, 9.0% had college degrees and 0.6% had post-graduate degrees.

**Table 2:** **Demographic characteristics of study participants (** ***N*** **= 122,058)**

***Category***	***Answer***	***N***	%
Age group (years)	25–34	27226	22.3
	35–44	40832	33.5
	45–54	27092	22.2
	55–64	19641	16.1
	≥65	7105	5.8
	Missing	162	0.1
	Mean (SD)	44.2 (11.6)	
Location	Urban	33696	27.6
	Rural	88362	72.4
Educational status	Primary or less	48750	39.9
	Middle	42483	34.8
	High	19186	15.7
	College	10932	9.0
	Post-graduate	707	0.6
Marital status	Married	117142	96.0
	Single	1464	1.2
	Widowed/divorced	3452	2.8
Annual household income (RMB)	<15000	70310	57.6
	≥15000	51748	42.4
Occupation	Peasant	67827	55.6
	Worker	21311	17.5
	Medical staff	2376	1.9
	Others	30544	25.0
Family history of breast cancer	No	120772	98.9
	Yes	1286	1.1
Smoking	No	121090	99.2
	Yes	968	0.8
Drinking	No	121642	99.7
	Yes	415	0.3

In our study, 22,740 women (18.6%) showed high awareness, and 99318 (81.4%) poor awareness. Table 1 summarizes their responses to questions about breast cancer; 98,362 (80.6%) knew that breast cancer was a common cancer among women, 86,405 (70.8%) thought breast screening was helpful in early detection of breast cancer, and 86,165 (70.6%) believed that early detection of breast cancer could improve survival. Of risk factors for breast cancer, 38,479 (31.5%) knew that family history of breast cancer is a risk factor for breast cancer; and 13,727 (11.2%) knew about menarche earlier than 12 years of age; 16,932 (13.9%) knew about nulliparity or late childbirth, 16,758 (13.7%) knew about later menopause, 23,347 (19.1%) knew about high-fat diets, 23,793 (19.5%) knew about long time drinking and 25,231 (20.7%) knew about long-term use of estrogen drugs.

**Figure 1:**
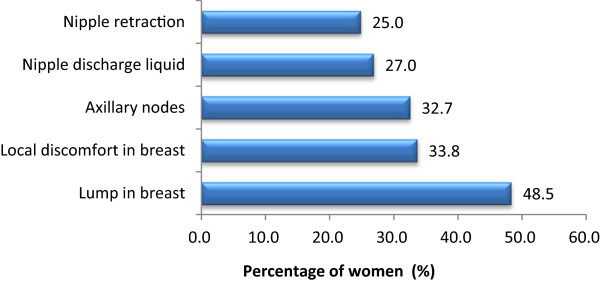
**Percentage of participants who identified each potential breast cancer symptom (N = 98,362).**

Among the women who knew what breast cancer was, nearly half (48.5%) were aware that a lump in the breast was a symptom of breast cancer (Figure 1). However, knowledge of other symptoms was generally poor; only 23.0% and 21.3%, respectively, recognized nipple discharge liquid and nipple retraction as signs of breast cancer.

As to breast screening and early detection awareness, 42178 of 122058 subjects (43.6%) had received breast examinations; including 26.5% who had ever carried out BSEs, 33.3% who received clinical breast examination, 17.5% who received breast ultrasound and 26.5% who received breast X-ray.

Results for univariate analysis at α = 0.05 indicated that awareness and knowledge of breast cancer were related age, location (urban or rural), education status, occupation, marital status, household annual income, family history of breast cancer, drinking, smoking, overall life satisfaction and behavioral prevention score. Multivariate analysis of these factors (α = 0.05) identified age, location, occupation, family history of breast cancer, household annual income, behavioral prevention score, smoking and drinking habits, and overall life satisfaction to independently correlate with breast cancer awareness (Table 3).

**Table 3:** **Multivariate analysis of factors related to knowledge of breast cancer**

	***B***	***S.E.***	***Wald***	***P***	***OR***	***95.0% CI***
Age	−0.025	0.008	11.126	0.001	0.975	0.960–0.990
Location	0.516	0.023	504.259	<0.0001	1.675	1.602–1.752
Family history of BC	0.210	0.072	8.645	0.003	1.234	1.073–1.420
*Occupation*						
Farmer				<0.0001	1.000*	
Worker	0.494	0.027	347.684	<0.0001	1.640	1.557–1.727
Medical staff	1.563	0.051	922.679	<0.0001	4.774	4.316–5.281
Other	0.839	0.026	1038.348	<0.0001	2.313	2.198–2.434
Household annual income	−0.873	0.022	1546.955	<0.0001	0.418	0.400–0.436
Behavioral prevention score	1.42	0.018	5941.213	<0.0001	4.137	3.991–4.290
Not smoking	0.748	0.179	17.517	0.039	2.113	1.488–2.999
Not drinking	0.355	0.172	4.261	<0.0001	1.427	1.018–2.000
Overall life satisfaction	−0.347	0.017	403.96	0.001	0.707	0.683–0.731

*Reference.

BC: breast cancer; CI: Confidence interval.

Location: urban or rural, reference: rural group.

Family history of BC, reference: those with no family history of BC.

Occupation: “Other” includes occupations such as teacher, civil servant, individual business, driver, service, company employee and housewife.

Household annual income, reference: low income group.

Behavioral prevention score was a cumulative score of 5 items; reference: low scores.

Not smoking, reference: smokers.

Not drinking, reference: drinkers.

Overall life satisfaction was a cumulative score of 12 items, reference: high life satisfaction.

Reliability and construct validity, and internal consistency reliability of the 15-item scale of knowledge about breast cancer and its subscales were assessed using reliability analysis (Cronbach’s alpha); the α-coefficient for the total scale was 0.910. The alpha coefficient >0.7 was considered acceptable for internal consistency reliability [21]. Exploratory factor analysis was conducted to explore construct validity. The Kaiser–Meyer–Olkin measure produced a coefficient of 0.87, indicative of excellent sampling adequacy. Bartlett’s test of sphericity produced a value of 1,298,838 (*P* < 0.001), indicating that the correlation matrix was unlikely to be an identity matrix and was therefore suitable for factor analysis [22].

## Discussion

Our survey indicated that Chinese women tend to know about some lump-related symptoms of breast cancer, but generally had poor awareness of other breast cancer-related information including risk factors and some atypical symptoms. Only 18.6% of women surveyed showed high awareness, while other 81.4% were poorly aware.

In this study, insufficient knowledge was mainly in two areas: symptoms of breast cancer other than lumps, and risk factors. Only 23.0% and 21.3%, respectively, recognized nipple discharge liquid and nipple retraction as signs of breast cancer. These results were in line with other studies, which also showed poor awareness of breast cancer symptoms. A large-scale, population-based survey involving various cancer types in the United Kingdom also showed poor awareness of early warning signs of cancer among British women [23, 24]. Grunfeld EA et al. showed only 38% of people realized that nipple retraction was a sign of breast cancer, and awareness of risk factors for breast cancer were even lower. Again, these results were similar to those of studies conducted in other countries [14, 25–28]. The similarity may be related to China’s evolving living standards and increased lay understanding of health topics. However, popular understanding of health issues, including breast cancer, is often inaccurate or piecemeal.

The results also indicated that the awareness and understanding of breast cancer is associated with age, occupation, educational level and family income. Women 25–35 years of age, with high education level and high annual family income tend to be more aware of this information. Generally, in China, younger women pay more attention to health care and tend to be better educated; our analysis showed collinearity between age and education (correlation coefficient: ^−^0.299, *P* < 0.000). As a result these women are more likely to be active learners and to access available information. Actually, some studies have shown that cancer awareness among older age groups was even worse [23, 29, 30]. In particular, although most breast cancer is diagnosed in women older than 35 years, and age is an important risk factor for breast cancer, awareness that age is a risk factor is generally poor, for breast cancer and some other types of cancer [23, 31–33]. These findings suggest that more public health education should be addressed to women older than 35 years [34].

A similar trend was found in other surveys with regard to socioeconomic status [30, 35]. As stated above, significant differences of awareness were observed among women in different occupations, for two possible reasons. First, different occupations call for different educational levels, which could include knowledge of health care; and second, women of different occupations preferred different health service resources, which might affect their understanding of breast cancer. For example, in our survey, medical personnel were more knowledgeable about breast cancer than farmers (OR=4.774), because professional educations of medical employees make it easier for them to access the relevant knowledge. These results were consistent with several other studies [13, 36].

Notably, we found that women with family histories of breast cancer were more aware of and knowledgeable about risk factors for breast cancer (OR = 1.234). Similar findings have been reported in some other studies [35, 37, 38].

Women who know more about breast cancer risk factors and mammography are more likely to receive breast cancer screening, which can lead to earlier diagnosis and treatment and improved survival. Our study found that the rate of detection of early-stage breast cancer was relatively low in eastern China—even lower than some survey results [13, 39]. Rates of BSE practice, clinical examination and X-ray screening were lower than the study of Whitman et al. (74%–90% had a mammogram). In our study, only 26.5% received breast X-rays. We thought lack of related knowledge might have led to lower rates of early detection. Interestingly, current study results also showed that women who previously had early screening had better knowledge scores (correlation coefficient: 0.213; *P* < 0.05), which indicates an effective interaction between awareness and screening. Although the investigators of the Shanghai study concluded that “intensive BSE instruction in the absence of mammography would be unlikely to reduce breast cancer mortality,” and the American Cancer Society no longer recommends that all women perform monthly BSE, most researchers agree that self-examination probably improves awareness and might play a part in nationwide programs for earlier-stage detection in China [40–42]. This result also implies that well-conducted health education programs lead to better health practices [43].

A limitation of this study is the lack of uniform standards and methods for measuring breast cancer-related knowledge. Here, we design a questionnaire of 15 questions to measure knowledge of breast cancer, which displayed high scale reliability (Cronbach's α value: 0.910).We will evaluate the scale’s reliability and validity in a future study.

## Conclusions

This study showed that most participants knew of breast cancer as a disease entity, but their awareness and understanding about the disease was very poor. As breast cancer incidence increases, a breast cancer awareness and education program is necessary and urgent, especially for women aged 35 years and older.

## References

[1] GLOBOCAN (2012). Estimated Cancer Incidence, Mortality and Prevalence Worldwide.

[2] Siegel R, Naishadham D, Jemal A (2013). Cancer statistics. CA Cancer J Clin.

[3] Chen W, Zheng R, Zhang S, Zhao P, Li G, Wu L, He J (2013). The incidences and mortalities of major cancers in China, 2009. Chin J Canc.

[4] Alteri R, Barnes C, Burke A, Gansler T, Gapstur S, Gaudet M, Kramer J, Newman LA, Niemeyer D, Richards C, Runowicz C, Saslow D, Simpson S, Smith R, Sullivan K, Wagner D, Xu JQ (2013). Breast Cancer Facts & Figures 2013–2014.

[5] Berry DA, Cronin KA, Plevritis SK, Fryback DG, Clarke L, Zelen M, Mandelblatt JS, Yakovlev AY, Habbema JD, Feuer EJ (2005). Effect of screening and adjuvant therapy on mortality from breast cancer. N Engl J Med.

[6] Rosmawati NH (2010). Knowledge, attitudes and practice of breast self-examination among women in a suburban area in Terengganu, Malaysia. Asian Pac J Canc Pre.

[7] Independent UK (2012). Panel on breast cancer screening: the benefits and harms of breast cancer screening: an independent review. Lancet.

[8] Nelson HD, Tyne K, Naik A, Bougatsos C, Chan B, Nygren P, Humphrey L (2009). Screening for Breast Cancer: Systematic Evidence Review Update for the US Preventive Services Task Force.

[9] Tabar L, Vitak B, Chen TH, Yen AM, Cohen A, Tot T, Chiu SY, Chen SL, Fann JC, Rosell J, Fohlin H, Smith RA, Duffy SW (2011). Swedish two-county trial: impact of mammographic screening on breast cancer mortality during 3 decades. Radiology.

[10] Paci E (2012). Euroscreen working group: summary of the evidence of breast cancer service screening outcomes in Europe and first estimate of the benefit and harm balance sheet. J Med Screen.

[11] Gotzsche PC, Jorgensen KJ (2013). Screening for breast cancer with mammography. Cochrane Database Syst Rev.

[12] Howlader N, Noone AM, Krapcho M, Garshell J, Miller D, Altekruse SF, Kosary CL, Yu M, Ruhl J, Tatalovich Z, Mariotto A, Lewis DR, Chen HS, Feuer EJ, Cronin KA: *SEER Cancer Statistics Review, 1975–2011*. Bethesda, MD: National Cancer Institute; http://seer.cancer.gov/csr/1975_2011/, based on November 2013 SEER data submission, posted to the SEER web site, April 2014

[13] Chen Y, Xia HO, Oakley D, Jia HL, Deng W (2007). The survey for awareness of breast cancer and early screening in Shanghai Women. Shanghai Nursing.

[14] Guvenc I, Guvenc G, Tastan S, Akyuz A (2012). Identifying women's knowledge about risk factors of breast cancer and reasons for having mammography. Asian Pac J Canc Prev.

[15] Fan L, Strasser-Weippl K, Li JJ, St Louis J, Finkelstein DM, Yu KD, Chen WQ, Shao ZM, Goss PE (2014). Breast cancer in China. Lancet Oncol.

[16] *The notice of “Two-cancer in rural women in China 2010” of the Ministry of Health and Women's Federations*. http://www.nhfpc.gov.cn/fys/s3581/201007/02a20b251ac646f2896dbc0b71a2cf92.shtml

[17] Lurie N, Margolis KL, McGovern PG, Mink PJ, Slater JS (1997). Why do patients of female physicians have higher rates of breast and cervical cancer screening. J Gen Intern Med.

[18] Bekker H, Morrison L, Marteau TM (1999). Breast screening: GPs' beliefs, attitudes and practices. Fam Pract.

[19] Okobia MN, Bunker CH, Okonofua FE, Osime U (2006). Knowledge, attitude and practice of Nigerian women towards breast cancer: a cross-sectional study. World J Surg Oncol.

[20] Akhigbe AO, Omuemu VO (2009). Knowledge, attitudes and practice of breast cancer screening among female health workers in a Nigerian urban city. BMC Cancer.

[21] Streiner DL, Norman GR (2003). Health Measurement Scales: A Practical Guide to Their Development and Use.

[22] Tabachnick BG, Fidell LS (2001). Using Multivariate Statistics.

[23] Linsell L, Burgess CC, Ramirez AJ (2008). Breast cancer awareness among older women. Br J Cancer.

[24] Grunfeld EA, Ramirez AJ, Hunter MS, Richards MA (2002). Women's knowledge and beliefs regarding breast cancer. Br J Cancer.

[25] Robb KA, Miles A, Campbell J, Evans P, Wardle J (2006). Can cancer risk information raise awareness without increasing anxiety: a randomized trial. Prev Med.

[26] Oluwatosin OA, Oladepo O (2006). Knowledge of breast cancer and its early detection measures among rural women in Akinyele Local Government Area, Ibadan, Nigeria. BMC Cancer.

[27] Wu TY, Ronis D (2009). Correlates of recent and regular mammography screening among Asian-American women. J Adv Nurs.

[28] Vahabi M (2011). Knowledge of breast cancer and screening practices among Iranian immigrant women in Toronto. J Community Health.

[29] Adlard JW, Hume MJ (2003). Cancer knowledge of the general public in the United Kingdom: survey in a primary care setting and review of the literature. Clin Oncol.

[30] Webster P, Austoker J (2006). Women's knowledge about breast cancer risk and their views of the purpose and implications of breast screening–a questionnaire survey. J Publ Health.

[31] Waller J, McCaffery K, Wardle J (2004). Measuring cancer knowledge: comparing prompted and unprompted recall. Br J Psychol.

[32] Moser K, Patnick J, Beral V (2007). Do women know that the risk of breast cancer increases with age. Br J Gen Pract.

[33] Wardle J, Waller J, Brunswick N, Jarvis MJ (2001). Awareness of risk factors for cancer among British adults. Publ Health.

[34] Yu ZG, Jia CX, Liu LY, Geng CZ, Tang JH, Zhang J, Zhang Q, Li YY, Ma ZB (2012). The prevalence and correlates of breast cancer among women in Eastern China. PLoS One.

[35] Erbil N, Bolukbas N (2012). Beliefs, attitudes, and behavior of Turkish women about breast cancer and breast self-examination according to a Turkish version of the Champion Health Belief Model Scale. Asian Pac J Canc Prev.

[36] Zhao L, Li SJ, Wang T (2008). The survey for knowledge, attitude andbehavior of breast cancer screening among Beijing women]. Chin Publ Health.

[37] Tastan S, Iyigun E, Kilic A, Unver V (2011). Health beliefs concerning breast self-examination of nurses in Turkey. Asian Nursing Res.

[38] Dundar PE, Ozmen D, Ozturk B, Haspolat G, Akyildiz F, Coban S, Cakiroglu G (2006). The knowledge and attitudes of breast self-examination and mammography in a group of women in a rural area in western Turkey. BMC Cancer.

[39] Whitman S, Shah AM, Silva A, Ansell D (2007). Mammography screening in six diverse communities in Chicago–a population study. Cancer Detect Prev.

[40] Thomas DB, Gao DL, Ray RM, Wang WW, Allison CJ, Chen FL, Porter P, Hu YW, Zhao GL, Pan LD, Li W, Wu C, Coriaty Z, Evans I, Lin MG, Stalsberg H, Self SG (2002). Randomized trial of breast self-examination in Shanghai: final results. J Natl Cancer Inst.

[41] Plesnicar A, Golicnik M, Fazarinc IK, Kralj B, Kovac V, Plesnicar BK (2010). Attitudes of midwifery students towards teaching breast-self examination. Radiol Oncol.

[42] Austoker J (2003). Breast self examination. BMJ.

[43] Doshi D, Reddy BS, Kulkarni S, Karunakar P (2012). Breast self-examination: knowledge, attitude, and practice among female dental students in Hyderabad City, India. Indian J Palliat Care.

[CR44] The pre-publication history for this paper can be accessed here:http://www.biomedcentral.com/1471-2458/14/1004/prepub

